# *CER1* gene variations associated with bone mineral density, bone markers, and early menopause in postmenopausal women

**DOI:** 10.1186/1479-7364-7-21

**Published:** 2013-10-18

**Authors:** Theodora Koromila, Panagiotis Georgoulias, Zoe Dailiana, Evangelia E Ntzani, Stavroula Samara, Chris Chassanidis, Vassiliki Aleporou-Marinou, Panagoula Kollia

**Affiliations:** 1 Laboratory of Human Genetics, Department of Genetics & Biotechnology, Faculty of Biology, National and Kapodistrian University of Athens, Athens 15701, Greece; 2 Department of Nuclear Medicine, University Hospital of Larissa, School of Medicine, University of Thessaly, Larissa 41110, Greece; 3 Department of Orthopaedic Surgery, School of Medicine, University of Thessaly, Larissa 41110, Greece; 4 Department of Hygiene and Epidemiology, University of Ioannina School of Medicine, Ioannina 45110, Greece

**Keywords:** *CER1*, *DKK1*, SNPs, Bone markers, Fracture, Menopause

## Abstract

**Background:**

Osteoporosis has a multifactorial pathogenesis characterized by a combination of low bone mass and increased fragility. In our study, we focused on the effects of polymorphisms in *CER1* and *DKK1* genes, recently reported as important susceptibility genes for osteoporosis, on bone mineral density (BMD) and bone markers in osteoporotic women. Our objective was to evaluate the effect of *CER1* and *DKK1* variations in 607 postmenopausal women. The entire *DKK1* gene sequence and five selected *CER1* SNPs were amplified and resequenced to assess whether there is a correlation between these genes and BMD, early menopause, and bone turnover markers in osteoporotic patients.

**Results:**

Osteoporotic women seem to suffer menopause 2 years earlier than the control group. The entire *DKK1* gene sequence analysis revealed six variations. There was no correlation between the six *DKK1* variations and osteoporosis, in contrast to the five common *CER1* variations that were significantly associated with BMD. Additionally, osteoporotic patients with rs3747532 and rs7022304 *CER1* variations had significantly higher serum levels of parathyroid hormone and calcitonin and lower serum levels of osteocalcin and IGF-1.

**Conclusions:**

No significant association between the studied *DKK1* variations and osteoporosis was found, while *CER1* variations seem to play a significant role in the determination of osteoporosis and a potential predictive role, combined with bone markers, in postmenopausal osteoporotic women.

## Introduction

Osteoporosis is a complex multifactorial disease characterized by low bone mass with a consequent increase in bone fragility, especially in the hips, spine, and wrist [1]. According to evidence arising from large observational studies [2,3] that is already part of the World Health Organization (WHO) and European guidelines for the management of osteoporosis, the clinical significance of osteoporosis is its established association with fracture risk, which is also mediated by a number of other epidemiological and clinical factors [4]. Apart from these traditional risk factors and due to a knowledge gap regarding fracture susceptibility, various bone-related biomarkers have also been proposed as potential fracture risk factors [5,6]. Several bone markers are measured in the serum in order to evaluate the bone turnover and to predict the fracture risk in elderly women [7,8].

The recently evolved novel concept of fat-bone interactions suggests that adipose tissue might profoundly affect bone formation and/or resorption [9]. Adipokines such as leptin have recently emerged as mediators of the protective effects of fat on bone tissue [10,11]. Moreover, serum osteocalcin (OC) has been considered as a specific marker of osteoblast function since OC levels correlate with bone formation rates. Insulin-like growth factor-1 (IGF-1) is also essential for the development and growth of the skeleton and maintenance of bone mass. IGF-1 promotes chondrogenesis and increases bone formation by regulating the functions of differentiated osteoblasts [12]. Furthermore, parathyroid hormone (PTH) is an important regulator of bone turnover because of the indirect stimulation of bone resorption through osteoclasts. While PTH increases the concentration of calcium in the blood, calcitonin (CT) reduces blood calcium and inhibits osteoclast activity in the bone.

In recent years, numerous gene polymorphisms (single nucleotide polymorphisms (SNPs)) have been associated with bone mineral density (BMD) and/or risk of fracture, identified either by a candidate gene approach or by genome-wide association studies (GWAS) [13,14]. The transforming growth factor beta (TGFbeta) and Wnt signaling pathways have a functional role in bone mass regulation, influencing both osteoblasts and osteoclasts.

The dickkopf Wnt signaling pathway inhibitor 1 (*DKK1*) gene in humans is located in 10q11.2 (NM_012242.2). The *DKK1* gene belongs to a small gene family of four members (*DKK1–4*) that encodes secreted proteins that typically inhibit canonical Wnt signaling by binding to the receptors of two different families, namely LRP5-LRP6 [15] and Kremen 1-Kremen 2 [16]. The extracellular regions of LRP5-LRP6 interact with the Wnt antagonists DKK1 and sclerostin (SOST). In molecular network analyses, SOST shows a strong, positive correlation with DKK1 [17,18]. Mice overexpressing Dkk1 develop severe osteopenia, in part due to diminished bone formation [19]. Finally, overexpression of DKK1 in glucocorticoid-induced osteoporosis [18,20,21] as well as in osteosarcoma and osteolytic metastatic bone disease in multiple myeloma [22-24] led to the hypothesis that *DKK1* is a strong candidate gene for the regulation of bone homeostasis.

Additionally, bone morphogenetic proteins (BMPs) are multifunctional growth factors that belong to the TGFbeta superfamily and have a significant role in bone remodeling. The activity of BMPs is controlled at different molecular levels [25]. A series of BMP antagonists bind BMP ligands and inhibit BMP functions. The human cerberus 1, DAN family BMP antagonist gene (*CER1*; NM_005454.2), a candidate gene for osteoporosis located in 9p23-p22, belongs to a distinct group of BMP antagonists (ligand traps) that can bind directly to BMPs and inhibit their activity [26-33].

In this case–control study, the whole *DKK1* gene sequence was replicated for the first time, as a possible regulator of bone mass as previously reported on GWAS [14]. Furthermore, the five common genetic variations of the *CER1* gene previously reported by Koromila et al. [32] were verified in a larger cohort. The correlation among the aforementioned SNPs with BMD, osteocalcin, and some bone turnover regulators as well as with menopause age of Greek postmenopausal women revealed significant conclusions.

## Results

### General characteristics of the assessed cohort

We analyzed 457 osteoporotic and 150 healthy postmenopausal women. As expected, the two groups revealed a statistically significantly difference (*p* < 0.001) in the mean *T*-score and the fracture record. The two groups were found to be similar in their other general characteristics, with the exception of mean years since menopause (*p* < 0.05). The majority of the osteoporotic group (78.9%) suffered from at least one fracture (vertebral, hip, or other fractures). Further details of both the osteoporotic and control groups are presented in Table 1.

**Table 1:** **Characteristics of the osteoporotic (*N *= 457) and control (*N *= 150) groups**

	**Control**	**Osteoporotic**
Age (years), mean [SD]	68.3 [11.2]	70.1 [11.3]
BMI (kg/m^2^), mean [SD]	28.1 [5.3]	26.9 [5]
Smoking (%)		
No	80.3	82.7
Yes	19.7	16.3
Years since menopause, mean [SD]	18.1 [11.7]	21.0 [12]
*T*-score, mean [SD]	−0.6 [0.3]	−2.8 [0.6]
Vertebral fracture (%)		
No	100	88.3
Yes	0	11.7
Hip fracture (%)		
No	100	56.2
Yes	0	43.8
Other fractures (%)		
No	100	76.6
Yes	0	23.4

### *DKK1* and *CER1* gene variants

The analysis of the whole *DKK1* gene sequence revealed six SNPs. Among the *DKK1* SNPs, rs11001560, rs11815201, rs112910014, and rs1569198 are intron-located; rs74711339 is located in the 3′ untranslated region (UTR); and the synonymous variation rs2241529 is located in exon 2. No significant association for the identified *DKK1* variants and BMD was found. Moreover, we found no significant association between *DKK1* and age, body mass index (BMI), smoking, early menopause, or bone markers.

Genotype distributions of all *CER1* alleles were in Hardy-Weinberg equilibrium (*p* < 0.05). Although, among the five *CER1* SNPs, rs3747532 and rs1494360 are not independent ones (*r*^2^ > 0.8) while the other three SNPs are not on any array according to SNAP analysis, we observed a statistically significant association for all five *CER1* SNPs (Table 2). Specifically, the rs1494360 SNP was independently associated with hip fractures (*p* = 0.043) or the presence of any fracture (*p* < 0.01) when multiple logistic regression analysis was performed for the prediction of fractures in the osteoporotic patients from the *CER1* sequence variations, adjusted for age, sex, smoking, BMI, years since menopause, and calcium intake, confirming our previous report [32]. Homozygotes or heterozygotes for the above SNP were at a higher risk of hip fracture (1.98-fold) and any fracture (1.38-fold). On the other hand, no significant association between *DKK1* and BMD, age, BMI, smoking, years since menopause, calcium intake, or fracture was found.

**Table 2:** **Association of *CER1 *genotypes with *T*-score and multiple logistic regression analysis for fracture prediction**

***CER1 *genotypes**	**Total cohort**			**Osteoporotic patients**					
	***T*-score**			**Vertebral fracture**		**Hip fracture**		**Any fracture**	
	**Mean**	**SD**	***P***	**OR (95% CI)**	***P***	**OR (95% CI)**	***P***	**OR (95% CI)**	***P***
rs3747532: (C/C)	−1.1	1.6	*<0.05*	1.71 (0.2–3.13)	NS	1.63 (0.76–3.48)	NS	1.79 (0.79–4.09)	NS
(C/G), (G/G)	−2.0	1.0							
rs1494360: (G/G)	−1.1	1.4	*<0.05*	2.12 (0.23–13.54)	NS	*1.98* (0.3–23.22)	*<0.05*	*1.38* (0.49–15.5)	*<0.01*
G/T), (T/T)	−2.4	1.1							
rs7022304: (A/A)	−1.1	1.5	*<0.05*	0.90 (0.23–3.49)	NS	1.22 (0.49–3.01)	NS	1.47 (0.49–8.1)	NS
(A/G), (G/G)	−2.2	1.2							
rs17289263: (A/A)	−1.0	1.4	*<0.05*	1.55 (0.42–5.04)	NS	1.15 (0.48–2.75)	NS	2.18 (0.61–5.2)	NS
(A/G), (G/G)	−2.2	1.2							
rs74434454: (T/T)	−1.0	1.5	*<0.05*	1.13 (0.46–3.72)	NS	1.90 (0.34–10.56)	NS	1.98 (0.3–13.22)	NS
(T/C), (C/C)	−2.2	1.1							

Significant values are shown in italics; *NS* not significant.

### Bone markers

**Figure 1:**
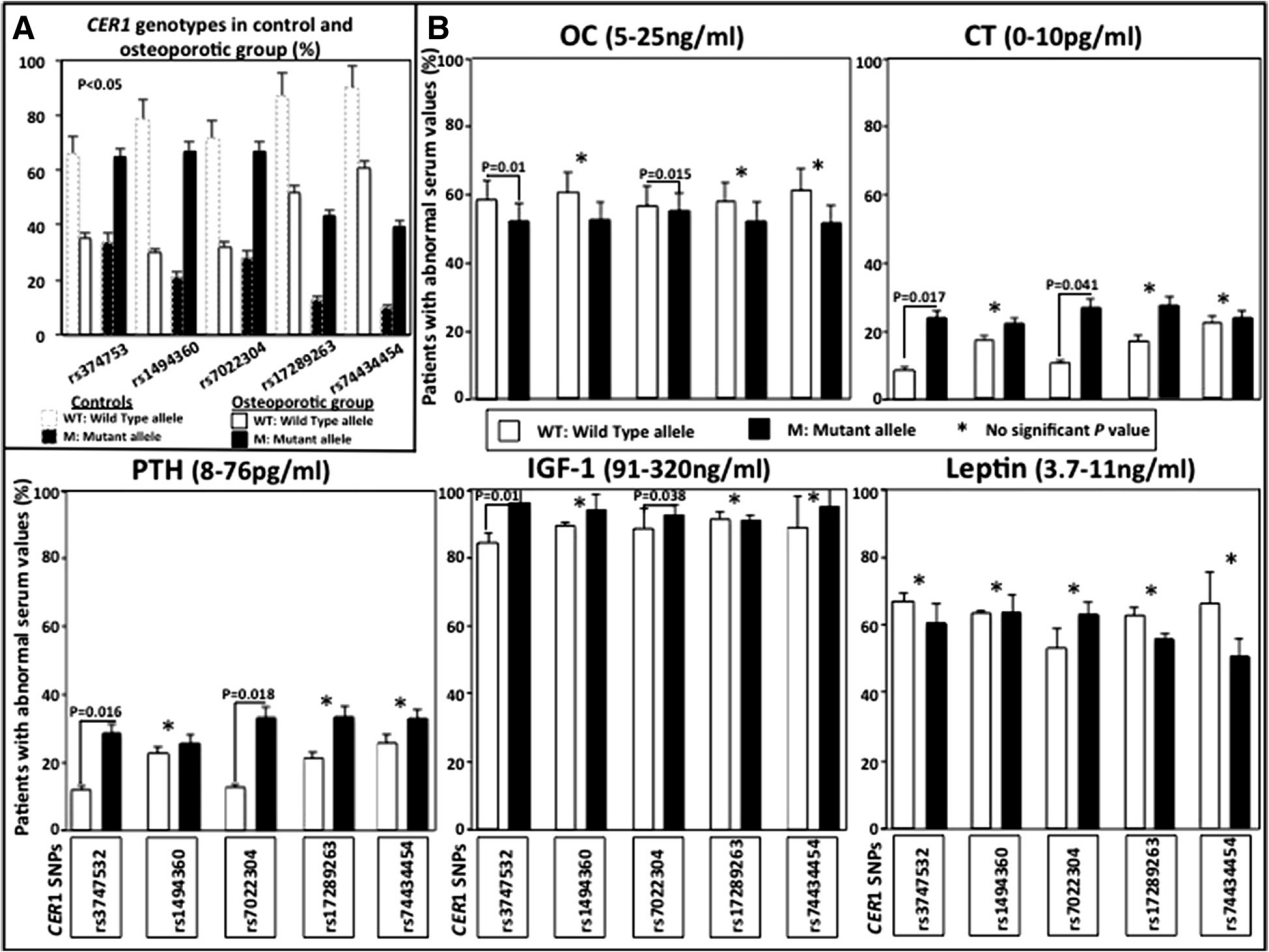
***CER1 *genotypes in postmenopausal women (A) and correlation with abnormal bone marker levels in osteoporotic patients (B).**

Among the studied bone markers previously referred, the serum levels of leptin did not change between osteoporotic patients and controls at any *CER1* variation. Compared to controls' values as well as to normal values' range per bone marker, a statistically significant number of osteoporotic patients with minor alleles of rs3747532 and rs7022304 had higher serum levels of PTH (mean = 78.4, standard deviation (SD) = 41.23) and CT (mean = 10.1, SD = 4.13) and lower serum levels of OC (mean = 4.9, SD = 3.52) and IGF-1 (mean = 80.2, SD = 62.62) (Figure 1). In addition, only serum OC levels and patients with hip fractures were significantly correlated and were found to be lower than total osteoporotic and control groups (*p* = 0.012), supporting the previous reports of Akesson et al. [34,35]. No significant association was found between the aforementioned bone markers and the age of menopause.

### Menopause

Postmenopausal women with osteoporosis seem to suffer menopause 2 years earlier than healthy women (*p* ≤ 0.05) as it is presented in Table 3. In addition, patients with hip fractures suffered menopause significantly earlier compared to the control group. However, our results did not verify an association between sequence variations of *DKK1* and *CER1* genes and bone marker serum levels or menopause age in the osteoporotic or in the total cohort group (osteoporotic and control).

**Table 3:** Menopause age and serum OC value correlation with control and osteoporotic (total, hip/vertebral fracture) groups

**Study group**	**Age of menopause**			**OC (5**–**25 ng/ml)**		
	**Mean**	**SD**	***P***	**Mean**	**SD**	***P***
Control	51.2	0.95	0.041	6.2	3.38	0.752
Osteoporotic total	49.1	12.33		5.4	4.75	
Osteoporotic hip fracture	48.6	5.85	0.024	4.7	4.57	0.012
Osteoporotic vertebral fracture	50.0	4.13	0.072	5.3	2.97	0.197

## Discussion

Most genetic studies on osteoporosis, until now, have focused on the regulation of BMD. A number of them suggest an important genetic component in the determination of peak bone mass and, in some instances, in the susceptibility to subsequent fractures.

In our study, we investigated the possible association of two important susceptibility genes for osteoporosis, *DKK1* and *CER1*, that participate in Wnt and TGFbeta signaling pathways, respectively, and are known for their functional role in bone mass regulation. The *DKK1* gene is able to modulate canonical Wnt signaling, and because of the established role of this pathway in the regulation of bone strength, this study aimed at understanding the influence of common genetic variations in *DKK1* and *CER1* genes on BMD, bone markers, and age of menopause. In a large genome-wide linkage scan, Ralston et al. [36] already suggested that the chromosomal region 10q21 containing the *DKK1* gene was specifically associated with the regulation of BMD in men.

Our findings for two *DKK1* variations, rs2241529 and rs1569198, support the previous report of Piters et al*.*[18] in the male population, while our report is the first in Caucasian women. In addition, the recent meta-analysis of GWAS of Estrada et al. revealed no correlation with any variation inside the *DKK1* gene sequence, although a variation upstream of the *DKK1* gene was significantly associated with FN-BMD (*p* = 1.3 × 10^−5^) and LS-BMD (*p* = 3.2 × 10^−4^) as well as with fractures [14].

Our previously reported findings in Caucasians [32] as well as the report of Tang et al. in southern Chinese women [33] suggest a significant association between *CER1* variations and BMD and/or fragility risk. Among all *CER1* sequence variations studied, only the rs3747532 SNP, located in exon 1, results in an Ala>Gly amino acid change, but both amino acids are classified as nonpolar. Both rs1494360 and rs7022304 SNPs are located in introns, rs74434454 is located in the 3′UTR, and the synonymous rs17289263 SNP is located in exon 2. Moreover, mice studies suggested that the *CER1* gene is an inhibitor of BMPs. BMP signaling is very important in bone development; it is not surprising that variations in BMP antagonists may affect skeletogenesis and BMD variations in humans (e.g., the sclerosteosis/van Buchem disease gene, which is caused by mutations in SOST) [37].

This is the first report on the correlation of rs3747532, rs1494360, rs7022304, rs17289263, and rs74434454 *CER1* variations with early menopause and bone markers. When *CER1* variations were correlated with the age of menopause, they were found to be independent while osteoporotic women with hip fracture were found to suffer menopause approximately 2.5 years earlier than the control group. Osteoporotic patients with rs3747532 or rs7022304 *CER1* variations were found to have significantly higher serum levels of PTH and CT, compared to both controls' and normal values per bone marker. Higher PTH levels in osteoporotic patients are in accordance with the indirect stimulation of bone resorption by PTH through osteoclasts. A further pharmacogenomic analysis of the above variations with different osteoporotic treatments could be of great interest in order to understand their mechanism. Both rs3747532 and rs7022304 variations were associated with low levels of OC and IGF-1 in osteoporotic postmenopausal women. Furthermore, low serum values of OC were associated with osteoporotic hip fractures, concluding that bone formation, as assessed by OC, is apparently lower in elderly women who sustain a hip fracture. Follow-up measurements in osteoporotic patients' serum samples, after 6 months and 1 year of fracture or starting therapy, will possibly show a stronger correlation with the *CER1* gene, leading to a new insight into personalized therapy of osteoporosis.

## Conclusions

Our study underlines a significant association of two sequence variations of the *CER1* gene with PTH, CT, OC, and IGF-1 in a Hellenic cohort of postmenopausal women. The studied *DKK1* SNPs seem to have no correlation with either the bone markers or the age of menopause, while the association of the *CER1* gene with bone markers supports its previously reported correlation with osteoporosis and suggests its potential role as a predictive marker of osteoporosis and hip fracture in postmenopausal women. In further GWAS, both the studied *CER1* and *DKK1* variations should be included in order to evaluate their biological role in osteoporosis.

## Methods

### Subjects

In this case–control study, peripheral blood samples were collected from 700 postmenopausal Greek women, who were treated at the Department of Orthopaedic Surgery of the University Hospital of Thessalia in Larissa, Greece, and gave their informed consent prior to their inclusion in the study. All the subjects of the present study underwent a physical examination and were interviewed using a structured questionnaire to obtain information on age, BMD, age of menopause, fracture, family history of osteoporosis and fracture, medical and reproductive history, smoking, alcohol intake, physical activity, and other secondary causes. Subjects were excluded from this study if they had diseases known to affect bone metabolism, were premature to menopause (absence of menstruation for at least 12 months, age <45 years), or had a history of drug use that could affect bone turnover and BMD. Moreover, high-trauma fractures including major trauma occurring during a motor vehicle accident or a fall from more than the standing height were excluded. Therefore, only 655 postmenopausal women met the inclusion criteria, of which 457 individuals were osteoporotic and 150 were normal, according to their dual-energy X-ray absorptiometry (DXA) findings (Table 1). In order to avoid misclassification and a potential effect dilution, 48 subjects with ‘gray-zone’ *T*-scores ranging between −1 and −2.5 were excluded from the study; thus, the study included 607 subjects. The study was approved by the Ethics Committee of the University of Thessalia, Larissa, Greece, and conducted according to the Declaration of Helsinki.

### *T*-score

BMD was measured at the femoral neck and at the lumbar spine (L2 to L4) by DXA. Cases were defined as subjects with a low BMD (*T*-score ≤−2.5) at either the spine or the hip, which was equivalent to osteoporosis according to the WHO definition [38]; control subjects were individuals with normal BMD (*T*-score >−1) without a history of fracture.

### Bone markers

Patients and controls were fasted for at least 12 h. Venous blood samples were drawn in the morning between 8:00 and 9:00 a.m., and patients' samples were measured within a mean of 12 h (±5 h) of fracture and before starting treatment. The samples were immediately centrifuged and stored at −80°C for further analysis. Total serum leptin and IGF-1 levels were measured using human radioimmunoassay (RIA) diagnostic kits (KIPMR44 and KIP1588, respectively, DIASource Europe SA, Louvain-La-Neuve, Belgium). The leptin kit is suited for human leptin, and no cross-reactivity has been found with other proteins such as insulin or IGF-1. The sensitivity of the leptin assay is 0.1 ng/ml, with a calibrators' range of 0–64 ng/ml. The IGF-1 kit has a sensitivity of 3.4 ng/ml and a calibrators' range of 0–1,529 ng/ml, with no cross-reactivity to insulin and growth hormone. Serum human intact osteocalcin, parathyroid hormone, and calcitonin values were measured using human immunoradiometric assay (IRMA) diagnostic kits (KIP1381, KIP1491, and KIP0429, respectively, DIASource Europe SA, Louvain-La-Neuve, Belgium). The OC kit has a sensitivity of 0.22 ng/ml and a calibrators' range of 0–69 ng/ml, with no cross-reactivity to N-terminal and C-terminal fragments. The PTH kit has a sensitivity of 4.1 pg/ml and a calibrators' range of 0–973 pg/ml and does not cross-react with PTH fragments and PTH-related proteins. The CT kit has a sensitivity of 0.9 pg/ml and a calibrators' range of 0–674 pg/ml. No significant interference has been found (at concentrations up to 100 ng/ml) with calcitonin gene-related peptide (CGRP), salmon calcitonin, katacalcin (PDN-21), and pro-calcitonin N-terminal. Moreover, all RIA and IRMA kits are calibrated against valid international standards. The radiotracer used in all kits is iodine-125 (^125^I, half-life *t*_1/2_ 60 days, 35.5-keV gamma radiation, 27–32-keV X-rays, no beta radiation). All sample assays were performed in duplicate and were included in the same run for each biological parameter. If the difference between duplicate results of a sample was more than 5%, the sample assay was repeated, and the in-run coefficients of variation were 3.9% for leptin, 3.4% for IGF-1, 2.9% for OC, 3.1% for PTH, and 2.8% for CT. An automatic gamma counter (Cobra II/5010, Packard, Conroe, TX, USA) was used to count the radioactivity and calculate the results.

### Amplification and resequencing of the human *CER1 and DKK1* genes

**Figure 2:**
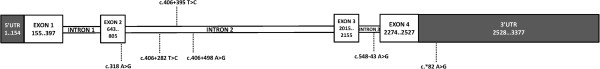
**Studied variations in the genomic structure of the *DKK1 *gene.**

Genomic DNA was isolated using QIAamp DNA Blood Mini Kit (QIAGEN, Venlo, Netherlands). *CER1* and *DKK1* genes were polymerase chain reaction (PCR)-amplified and resequenced to identify the underlying sequence variation. Eleven pairs of primers, four pairs for *CER1* and seven pairs for *DKK1* (Table 4), were designed in order to cover the five variants of the *CER1* gene previously reported by Koromila et al. [32] as well as the entire sequence of the *DKK1* gene (3,377 bp) (Figure 2). Sequencing was performed twice per sample (two independent PCR products) in both forward and reverse orientations. Genomic DNA information was obtained from GenBank wild-type sequences [*CER1*: chromosome 9, NC_000009.11 (14719731..14722715), MIM: 603777, ID: 9350; *DKK1*: chromosome 10, NC_000010.10 (54074041..54077417), MIM: 605189, ID: 22943]. Sequence variants were verified using the MegaBACE 1000 DNA Sequencing System (Amersham Biosciences, Piscataway, NJ, USA). Six variants, rs2241529, rs11001560, rs11815201, rs112910014, rs1569198, and rs74711339, in the *DKK1* gene were detected (Figure 2), and five common variants were analyzed in *CER1* by multiple sequence alignments using Chromas Lite 2.01 software and BLAST analysis in the cohort. Five common SNPs in the *CER1* gene, namely rs3747532 (c.194C>G, exon 1), rs1494360 (c.507+506G>T, intron), rs7022304 (c.508-182A>G, intron), rs17289263 (c.531A>G, exon 2), and rs74434454 (c.*121T>C, 3′UTR), were resequenced. Among these five *CER1* SNPs, only rs3747532, which is located in exon 1, causes an amino acid change from Ala to Gly. Six SNPs in *DKK1* (Figure 2) were analyzed as well through direct resequencing. Among the *DKK1* SNPs, only rs2241529 is exon-located (c.318A>G, exon 2) and causes an amino acid substitution, while rs11001560, rs11815201, rs112910014, and rs1569198 are located in introns and rs74711339 in 3′UTR. An association between *DKK1* variations and BMD could not be attempted in our dataset (osteoporotic and control).

**Table 4:** **Primers for PCR and sequencing of the *DKK1 *gene**

**Localization (nucleotide)**	**Forward primer**	**Reverse primer**
	**5′ → 3′ sequence**	**5′ → 3′ sequence**
1–490	GCAGAGCTCTGTGCTCCCT	ACCGCACACATTCAGCACG
396–1,043	AGGTGAGAGGGGTCGGGCAC	CGGAGGAAGATAAGGACCTC
963–1,470	CGCTGAAGTATCTTCATTGCA	GGAGACCTCTTTAGCTGTCT
1,407–2,010	AGCACAGATCCACTAACTT	GGAAGCAGGAAATAGTGATT
1,945–2,481	GCCACTGTCACAGCTGTTA	TGGTGATCTTTCTGTATCCG
2,427–2,965	CCAGCGTTGTTACTGTGGA	CCAAGAGATCCTTGCGTTC
2,950–3,377	CGCAAGGATCTCTTGGAATGA	TAGGTATTATTAATTTATTGG

### Statistical analysis

Continuous variables are presented as mean and SD, while categorical variables are presented as absolute and relative frequencies. The Hardy-Weinberg equilibrium (HWE) was assessed in the control samples by applying an exact test. Deviation from HWE was considered nominally statistically significant at the *p* < 0.05 level [39,40]. Genotype frequency differences between cases and controls were tested using unconditional logistic regression without any adjustments. Odds ratios (ORs) and 95% confidence intervals (CIs) were estimated under the log-additive model, using the major allele in the Greek control population as reference. Odds ratios thus represent the risk conferred per copy of the minor allele. Secondary analyses also examined recessive and dominant models of inheritance. Pearson's correlation coefficient (*r*) was used to estimate the correlations in minor allele frequencies between our study and the HapMap CEU population [26]. The overall correlation between ORs in the Greek population and the GWAS population where each SNP was first discovered was also calculated. The power of the study to detect ORs similar to those previously found in the GWAS, given the allele frequencies observed in the Greek population, was estimated at an *α* value of 0.05.

Statistical analyses were run in Stata, version 10.1 (College Station, TX, USA). *P* values for association are two-tailed and not adjusted for multiple comparisons since this is a replication effort for associations that already have robust statistical support. Student's *t* tests were used for the comparison of mean values between osteoporotic and control groups. Analyses were conducted using SPSS statistical software (version 17.0). Design and reporting follow the STREGA guidance [41].

## Abbreviations

BMD: bone mineral density; BMI: body mass index; BMP: bone morphogenetic protein; CER1: cerberus 1; CT: calcitonin; DKK1: dickkopf Wnt signaling pathway inhibitor 1; DXA: dual-energy X-ray absorptiometry; GWAS: genome-wide association studies; IGF-1: insulin-like growth factor-1; IRMA: immunoradiometric assay; NS: not significant; OC: osteocalcin; OR: odds ratio; PTH: parathyroid hormone; RIA: radioimmunoassay; SNP: single nucleotide polymorphism; SOST: sclerostin; TGFbeta: transforming growth factor beta; UTR: untranslated region; WHO: World Health Organization.

## Competing interests

The authors declare that they have no competing interests.

## Authors' contributions

TK and PK designed the experiments. ZD provided the human blood and tissue samples. PG carried out the analysis of bone markers. TK, SS, and CC collected the samples and clinical data. TK and EEN prepared the statistical analysis. TK, PK, ZD, and VAM prepared the manuscript. All authors read and approved the final manuscript.
